# Clinico-pathological factors associated with radioiodine refractory differentiated thyroid carcinoma status

**DOI:** 10.1007/s40618-024-02352-z

**Published:** 2024-04-05

**Authors:** L. Schubert, A. M. Mbekwe-Yepnang, J. Wassermann, Y. Braik-Djellas, L. Jaffrelot, F. Pani, G. Deniziaut, C. Lussey-Lepoutre, N. Chereau, L. Leenhardt, M- O. Bernier, C. Buffet

**Affiliations:** 1 Service des pathologies thyroïdiennes et tumorales endocrines, Sorbonne Université, Groupe de Recherche Clinique n°16, GRC Tumeurs Thyroïdiennes, AP-HP, Hôpital Pitié-Salpêtrière, 45-83 boulevard de l’Hôpital, 75013 Paris, France; 2 Laboratory of Epidemiology, Institut de Radioprotection et de Sureté Nucléaire, BP 17, 92262 Fontenay aux Roses, France; 3 Medical Oncology Department and Thyroid and Endocrine Tumors Department, Groupe de Recherche Clinique n°16, GRC Tumeurs Thyroïdiennes, AP-HP, Hôpital Pitié-Salpêtrière, 75013 Paris, France; 4 Pathology Department, Sorbonne Université, Groupe de Recherche Clinique n°16, GRC Tumeurs Thyroïdiennes, AP-HP, Hôpital Pitié-Salpêtrière, 75013 Paris, France; 5 Nuclear Medicine Department, Sorbonne Université, Groupe de Recherche Clinique n°16, GRC Tumeurs Thyroïdiennes, AP-HP, Hôpital Pitié-Salpêtrière, 75013 Paris, France; 6 PARCC-Inserm U970, 56 rue leblanc, 75015 Paris, France; 7 Department of Endocrine Surgery, Sorbonne Université, Groupe de Recherche Clinique n°16, GRC Tumeurs Thyroïdiennes, AP-HP, Hôpital Pitié-Salpêtrière, 75013 Paris, France; 8 Laboratoire d’Imagerie Biomédicale (LIB), Sorbonne Université, CNRS UMR 7371, INSERM U1146, Paris, France

**Keywords:** Risk factors, Differentiated thyroid carcinoma, Radioiodine refractory thyroid carcinoma, Distant metastases, Prediction score

## Abstract

**Purpose:**

Risk factors for developing radioiodine refractory thyroid cancer (RAIR-TC) have rarely been analyzed. The purpose of the present study was to find clinical and pathological features associated with the occurrence of RAIR-disease in differentiated thyroid cancers (DTC) and to establish an effective predictive risk score.

**Methods:**

All cases of RAIR-DTC treated in our center from 1990 to 2020 were retrospectively reviewed. Each case was matched randomly with at least four RAI-avid DTC control patients based on histological and clinical criteria. Conditional logistic regression was used to examine the association between RAIR-disease and variables with univariate and multivariate analyses. A risk score was then developed from the multivariate conditional logistic regression model to predict the risk of refractory disease occurrence. The optimal cut-off value for predicting the occurrence of RAIR-TC was assessed by receiver operating characteristic (ROC) curves and Youden’s statistic.

**Results:**

We analyzed 159 RAIR-TC cases for a total of 759 controls and found 7 independent risk factors for predicting RAIR-TC occurrence: age at diagnosis ≥ 55, vascular invasion, synchronous cervical, pulmonary and bone metastases at initial work-up, cervical and pulmonary recurrence during follow-up. The predictive score of RAIR-disease showed a high discrimination power with a cut-off value of 8.9 out of 10 providing 86% sensitivity and 92% specificity with an area under the curve (AUC) of 0.95.

**Conclusion:**

Predicting the occurrence of RAIR-disease in DTC patients may allow clinicians to focus on systemic redifferentiating strategies and/or local treatments for metastatic lesions rather than pursuing with ineffective RAI-therapies.

**Supplementary Information:**

The online version contains supplementary material available at 10.1007/s40618-024-02352-z.

## Introduction

Thyroid cancer (TC) is the most frequent endocrine cancer. Incidence rates of new cases have been rising over the last decades to reach 10,665 in France in 2018 [1]. Differentiated thyroid carcinoma (DTC) including in particular papillary thyroid carcinoma (PTC) and follicular thyroid carcinoma (FTC), represents 90% of all thyroid malignancies. Most DTC patients are successfully treated with total thyroidectomy, radioactive iodine (RAI) therapy and thyroid-stimulating hormone (TSH)-suppressive therapy [2]. However, distant metastases (DM) occur in 4–23% of cases, most often in the lung [3, 4]. One-third of distant metastatic patients achieve remission with RAI therapy [5]. The remaining two-thirds are initially or will become radioiodine refractory [5]. These radioiodine refractory (RAIR) cancers represent 2–5% of all TC [5, 6] and are mostly treated with first-line antiangiogenic tyrosine kinase inhibitors (TKIs) [7–9] for patients with progressive disease. As TKI can fail mainly due to acquired resistance with tumor escape and the necessity of drug withdrawal because of adverse effects, the concept of redifferentiation strategy has emerged [10]. The redifferentiation of TC, typically with a short course of molecular targeted therapies capable of restoring radioiodine sensitivity has been applied for advanced RAIR-TC but could also be used before postoperative RAI administration in selected patients [11, 12].

Thus, there is a growing necessity to determine the risk factors for DTC patients being or becoming RAIR to personalize their management.

Very few studies have focused specifically on this issue [13–16]. The aim of this study was to determine the clinical and pathological risk factors of developing RAIR-disease from a cohort of PTC and FTC patients treated in our center since 1990.

## Patients and methods

### Study design and patients

**Fig. 1:**
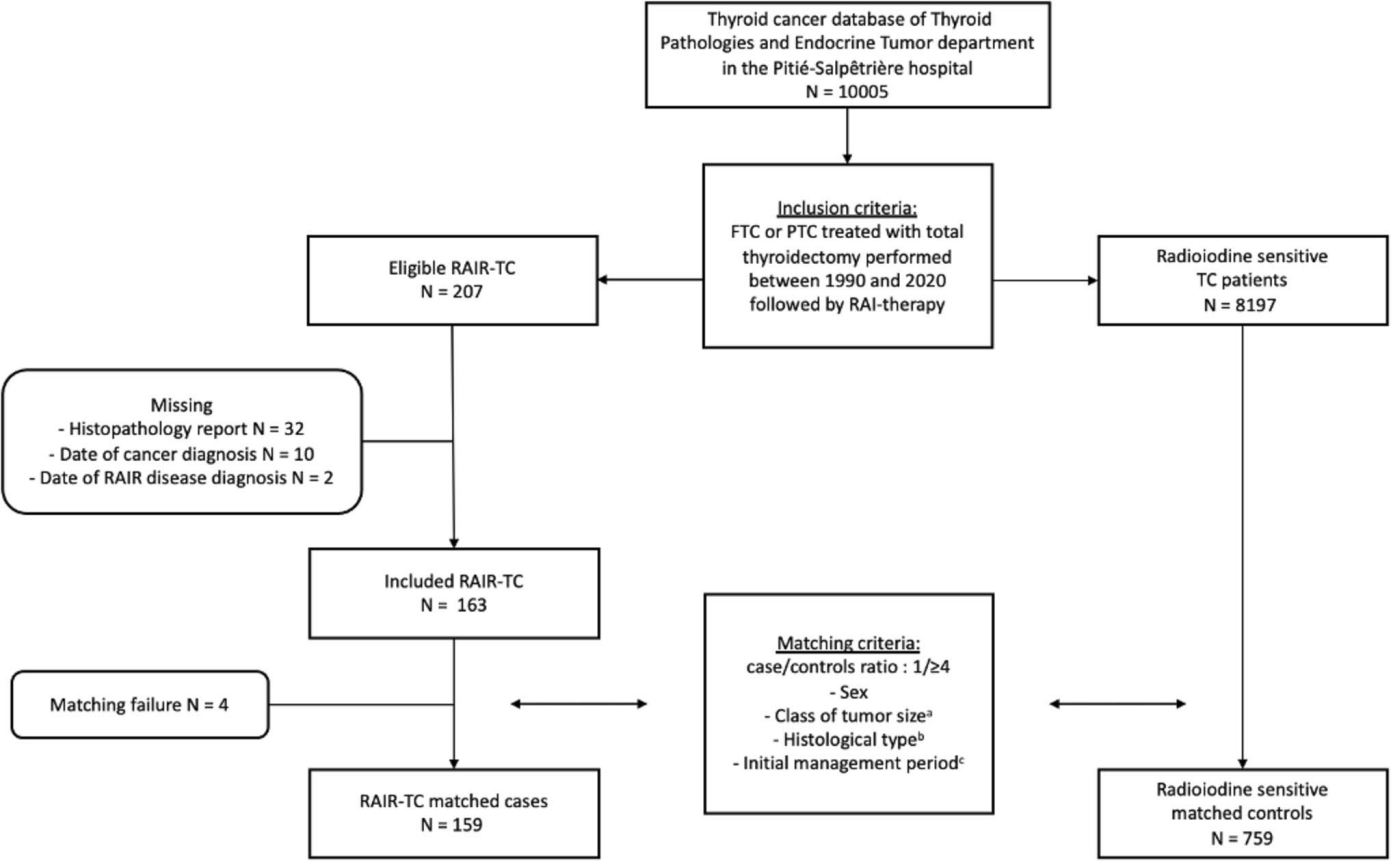
Flow chart of the cohort. **a** 20 mm, 20–40 mm, > 40 mm, **b** papillary thyroid cancer, follicular thyroid cancer, **c** 1990–2000, 2000–2010, > 2010

Our study was designed as a nested case–control study within the cohort of the 10.005 patients treated between 1990 and 2020 for TC at the Groupe Hospitalier Pitié-Salpêtrière (Paris, France). The cohort was filled prospectively from 1960 (APHP registry No. 2020011571338) based on medical records. Of the 10.005 patients with TC, 8.404 patients diagnosed with papillary or follicular TC were treated with radioiodine in the Department of Nuclear Medicine at the Groupe Hospitalier Pitié-Salpêtrière within the same period (Fig. 1). Written informed consent was obtained prospectively from each patient to use their clinical data. The study research was completed in accordance with the Declaration of Helsinki as revised in 2013. According to the French law, retrospective studies built exclusively on data, do not need the approval of “committees for the protection of individuals”.

### Identification of cases and controls

207 cases were identified as RAIR in the cohort according to the 2015 ATA Management Guidelines as follows [2]: metastatic disease that does not concentrate RAI at the time of the first or subsequent RAI treatment; RAI uptake retained in some lesions but not in others; metastatic disease that progresses despite the administration of a significant activity of RAI. Lastly, the following criterion, often used in clinical trials and highlighted by some learned societies [17, 18], was also used to define RAIR-TC: no remission despite a cumulative activity of RAI ≥ 600 mCi.

Patients with an unavailable histopathology report, date of cancer or RAIR-disease diagnosis were excluded (*n* = 44). The main cause was the absence of pathological report (*n* = 32) mainly because thyroid surgery was performed in a foreign country.

A total of 163 RAIR-TC patients were finally included. RAIR-TC patients were matched randomly with at least 4 radioiodine sensitive control TC patients based on the following criteria: (i) sex, (ii) primary histological tumor size (< 20 mm; 20–40 mm; > 40 mm), (iii) histological type (papillary; follicular), (iv) initial management period (1990–2000; 2000–2010; after 2010). These criteria were chosen to match patients on main known prognostic factors for DTC, i.e., sex, tumor size and histological type to individualize other risk factors. A formal computer-assisted randomization was used. Radioiodine sensitive controls are defined as patients who do not meet the definition of RAIR patients and are classified at the end of the follow-up: with excellent response, with biochemical incomplete/indeterminate response without morphological disease, with structural incomplete response but with RAI-avid lesions. The follow-up period was defined for the RAIR-TC cases as the period between the date of TC diagnosis and the date of RAIR-disease diagnosis. For the controls, the follow-up period was defined as the period between the date of TC diagnosis and the date of last visit. The follow-up period considered for controls was equal to, or greater than that of the matched RAIR-TC cases. Among the RAIR-TC included, four patients were not able to be matched due to the absence of appropriate controls and finally, 159 RAIR-TC cases were analyzed for a total of 759 controls.

### Management of thyroid cancer

Initial treatment included total thyroidectomy with or without lymph node dissection, completed by RAI therapy. Then, a post-therapeutic whole-body-scan (^131^I-WBS) was performed to enable the diagnosis of metastases and report their ^131^I-uptake status (metastatic location at initial work-up). All patients were subsequently subjected to levothyroxine treatment at a suppressive dose. During follow-up, the patients were regularly monitored by neck-ultrasound and blood samples (thyroglobulin, antithyroglobulin antibodies and TSH). Recurrence/persistence of distant metastases and their locations were defined by iodine-uptake on a diagnostic or therapeutic WBS and/or histological analysis and/or typical aspect on diagnostic imaging such as computed tomography (CT), 18-fluorodeoxyglucose (18F-FDG) positron emission tomography (PET), and/or magnetic resonance imaging.

### Statistical analysis

For descriptive analysis, quantitative variables were compared between the cases and the controls via the Wilcoxon test. Qualitative variables were compared using the Fisher test or the Pearson’s chi-squared test when applicable.

Conditional logistic regression was used to examine the association between refractory thyroid cancer and covariates. Covariates included: age; type of lymph node surgery; histopathological information: extrathyroidal extension defined as tumor extension into perithyroidal soft tissues or sternothyroid muscle, vascular invasion, multifocality (≥ 2 separate malignant foci), lymph node involvement; RAI uptake status after the first RAI treatment: cervical, pulmonary, bone and other metastatic locations; local or distant metastases during the follow-up (more than 6 months after the first RAI treatment): cervical, pulmonary, bone and other metastatic locations. Univariate analysis and then multivariate analysis using the lasso method to select the relevant variables were conducted. Adjusted odds ratios and 95% confidence estimates were calculated.

A prediction model was developed from the multivariate conditional logistic regression model to predict the risk of RAIR-disease occurrence. All variables selected by the Lasso method were introduced in our predictive model. From this model, a predictive score was derived by multiplying the predicted value given by the model by 10, thus obtaining a score varying from 0 to 10. Internal validation was performed. Discrimination and calibration were assessed on initial data. Then, the sample was split into two: one to develop the model and the other to validate the model. Discrimination and calibration were also performed on validation data. Bootstrap and *k*-fold cross-validation (*k* = 10) were applied.

The performance of the score was assessed by calculating the area under the receiver operating characteristic (ROC) curve (AUC) for discrimination, by calculating the maximum absolute calibration error for calibration and by calculating the Brier score (that included the component of discrimination and calibration).

To find the optimal cut-off for the classification of score values in the positive or the negative group, we used the Youden’s statistic, that is, sensitivity + specificity − 1. For each cut-off, the Youden’s statistic was computed and the chosen cut-off was the one that maximizes the Youden’s statistic. Sensitivity, specificity, positive predictive value and negative predictive value for the chosen cut-off were computed, as well as their confidence interval. *P* values below 0.05 were considered statistically significant. *R* software, v4.0.3 was used for analysis.

## Results

### Population characteristics and metastatic location during follow-up

The clinical and histological characteristics of the RAIR and control patients are compared in Table 1. The RAIR-DTC population included 159 patients for 759 controls. RAIR-TC patients were mostly females (62 and 65%, respectively). The median age was 62 years-old and 44 years-old (*P* < 0.001) with a mean time of follow-up of 6.8 ± 5.0 years and 10.5 ± 5.1 years, respectively, in RAIR-TC and radioiodine-sensitive TC groups. Time to RAIR-disease diagnosis ranged from 0.1 to 29.6 years (mean = 6.8 ± 5.0 years) and the criteria for diagnosing RAIR-disease were distributed as follows: 71% of patients had some or all metastases that does not concentrate RAI, 16% of patients had progressive disease despite the administration of a significant activity of RAI and 13% had no remission despite a cumulative activity of RAI ≥ 600 mCi.

**Table 1:** Patients baseline characteristics

Characteristics	RAI-sensitive TC group *n* = 759	RAIR-TC group *n* = 159	*P *value
Age at diagnosis—year	44 (34–56)	62 (51–69)	< 0.001
Age at diagnosis—year			< 0.001
< 55	554 (73%)	54 (34%)	
≥ 55	205 (27%)	105 (66%)	
Gender			0.54
Female	495 (65%)	99 (62%)	
Male	264 (35%)	60 (38%)	
Initial management period			0.79
1990–2000	126 (17%)	30 (19%)	
2000–2010	299 (39%)	61 (38%)	
>2010	334 (44%)	68 (43%)	
Criteria for RAIR classification			
No RAI uptake (in all or some metastases)		113 (71%)	
Progressive disease despite RAI		26 (16%)	
Cumulative activity of RAI ≥ 600 mCi		20 (13%)	
Histological analysis			
Histological type			0.81
FTC	205 (27%)	45 (28%)	
PTC	554 (73%)	114 (72%)	
Primary tumor size—mm	25 (12–45)	30 (10–45)	0.70
Primary tumor size			0.80
< 20 mm	282 (37%)	58 (36%)	
20–40 mm	267 (35%)	53 (33%)	
> 40 mm	210 (28%)	48 (30%)	
Multifocality	245 (32%)	53 (33%)	0.87
Extrathyroidal extension	174 (23%)	69 (43%)	< 0.001
Vascular invasion	144 (19%)	47 (30%)	0.004
Lymph node dissection	448 (59%)	107 (67%)	0.06
Lymph node metastasis	242 (32%)	85 (53%)	< 0.001

Data are median (interquartile range) or *n* (%)

FTC, follicular thyroid cancer; PTC, papillary thyroid cancer

Total thyroidectomy was performed in all patients, but lymph node dissection was carried out in 67% (*n* = 107) of the cases and 59% (*n* = 448) of the controls (*P* = 0.06). Regarding pathological characteristics, cases and controls were mostly PTC (72% and 73%, respectively). The median primary tumor size of the cases was 30 mm (IQR = 10–45) not significantly different from the controls (*P* = 0.7). Tumors were multifocal in the thyroid gland in 33% and 32% of the cases and controls, respectively (*P* = 0.87). Vascular invasion and extrathyroidal extension (ETE) were significantly more frequent in the cases with 30% and 43% of patients compared with the controls with 19% and 23% (*P* = 0.004 and *P* < 0.001, respectively). Moreover, neck lymph node metastases were more frequent in the cases than in the controls (53% vs. 32%; *P* < 0.001).

There was no difference in the matching criteria between the cases and the controls.

Whatever the metastatic location, metastases were more prevalent in the RAIR-TC patients than in the controls at initial work-up (Table 2): neck (14% vs. 4%, *P* < 0.001), lung (12% vs. 1%, *P* < 0.001), bone (8% vs. 0.3%, *P* < 0.001), and other location (4% vs. 1%, *P* = 0.03). Finally, follow-up metastases were also more prevalent in the RAIR-TC patients compared to the controls (Table 2): neck (59% vs. 14%, *P* < 0.001), lung (82% vs. 8%, *P* < 0.001), bone (26% vs. 2%, *P* < 0.001), and other locations (15% vs. 2%, *P* < 0.001).

**Table 2:** Metastatic location at initial work-up and during follow-up

Variable *n* (%)	RAI-sensitive TC group *n* = 759	RAIR-TC group *n* = 159	*P* value
Metastatic location at initial work-up			
Neck	28 (4%)	23 (14%)	< 0.001
Lung	7 (1%)	19 (12%)	< 0.001
Bone	2 (0.3%)	13 (8%)	< 0.001
Other	9 (1%)	6 (4%)	0.03
Metastatic location of persistence or recurrence during follow-up			
Neck	110 (14%)	94 (59%)	< 0.001
Lung	58 (8%)	131 (82%)	< 0.001
Bone	17 (2%)	42 (26%)	< 0.001
Other	12 (2%)	24 (15%)	< 0.001

### Variables associated with the development of RAIR-disease

Thirteen variables significantly associated with the development of RAIR-TC were identified in the univariate analysis (Table 3). In the multivariate analysis, the following seven variables remained significant: age at diagnosis ≥ 55 years [OR = 7.66; (CI 3.20–18.35), *P* < 0.001]; vascular invasion [OR = 4.40; (CI 1.60–12.07), *P* = 0.004]; synchronous metastases at initial work-up in neck [OR = 26.97; (CI 4.48–162.46), *P* < 0.001], lung [OR = 4.70; (CI 1.01–21.94), *P* = 0.049], and bone [OR = 1485.29; (CI 23.71–93059), *P* < 0.001]; recurrence or persistence during follow-up in neck [OR = 7.89; (CI 3.21–19.38), *P* < 0.001] and lungs [OR = 52.73; (CI 18.49–150.37), *P* < 0.001].

**Table 3:** Univariate and multivariate logistic regression analyses for risk factors of radioiodine refractory status

Characteristics	Univariate analysis		Multivariate analysis	
	OR (95% CI)	*P* value	adjusted OR (95% CI)	*P* value
Age at diagnosis (years)				< 0.001
< 55	1 Reference		1 Reference	
≥ 55	5.63 (3.84–8.27)	< 0.001	7.66 (3.20–18.35)	
Lymph node dissection	1.51 (1.03–2.21)	0.040	0.57 (0.23–1.40)	0.218
pN1	2.92 (1.99–4.28)	< 0.001	2.23 (0.91–5.46)	0.079
Multifocality	1.06 (0.73–1.54)	0.760	1.35 (0.61–3.02)	0.460
Vascular invasion	1.92 (1.23–2.99)	0.004	4.40 (1.60–12.07)	0.004
Extrathyroidal extension	3.00 (2.03–4.44)	< 0.001	0.85 (0.40–1.80)	0.666
Metastatic location at initial work-up				
Cervical	8.57 (3.86–18.99)	< 0.001	26.97 (4.48–162.46)	< 0.001
Lung	13.63 (5.41–34.29)	< 0.001	4.70 (1.01–21.94)	0.049
Bone	28.87 (6.49–128.43)	< 0.001	1485.29 (23.71–93059)	< 0.001
Other	3.28 (1.17–9.23)	0.020	11.14 (0.79–156.30)	0.074
Metastatic persistence or recurrence during follow-up				
Cervical	9.99 (6.46–15.46)	< 0.001	7.89 (3.21–19.38)	< 0.001
Lung	54.42 (27.60–107.30)	< 0.001	52.73 (18.49–150.37)	< 0.001
Bone	13.15 (7.16–24.17)	< 0.001	1.78 (0.50–6.32)	0.372
Other	15.14 (6.49–35.33)	< 0.001	0.34 (0.07–1.66)	0.181

### Scoring system for predicting the occurrence of radioiodine-refractory disease

**Fig. 2:**
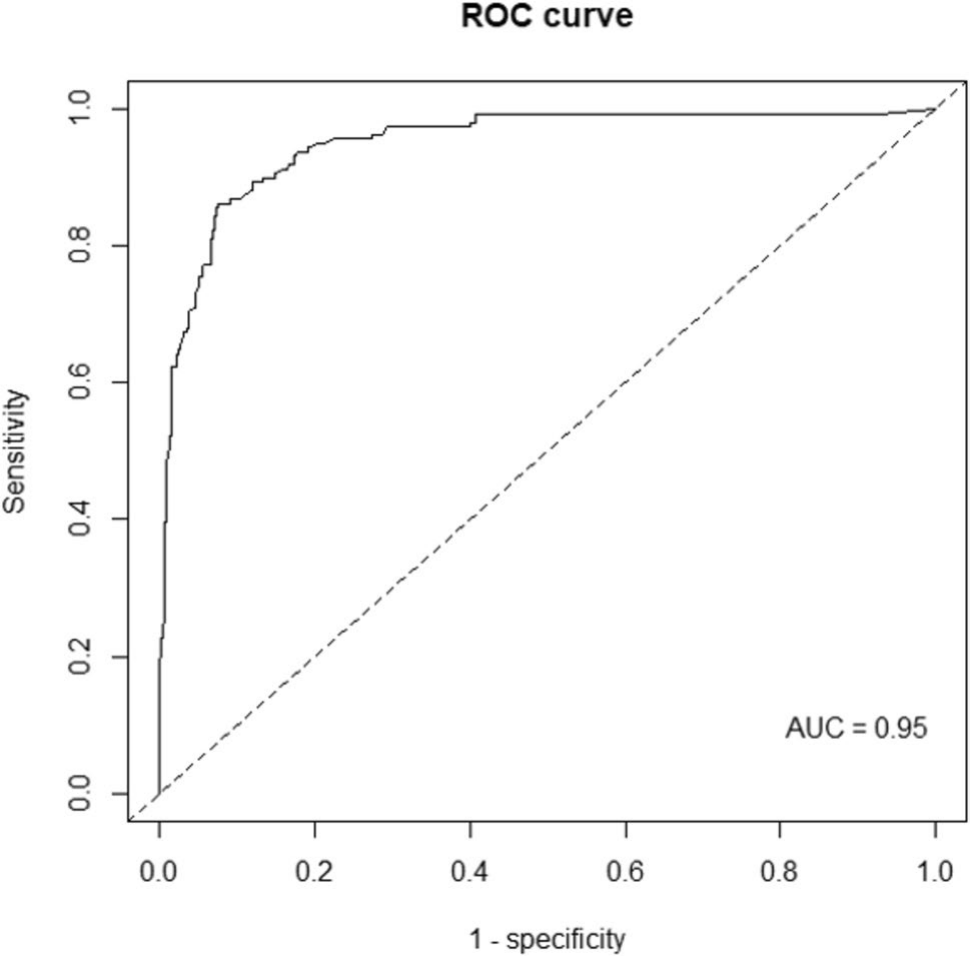
Receiver operating characteristics (ROC) curve of the scoring system to predict the occurrence of radioiodine refractory (RAIR) cancer

**Fig. 3:**
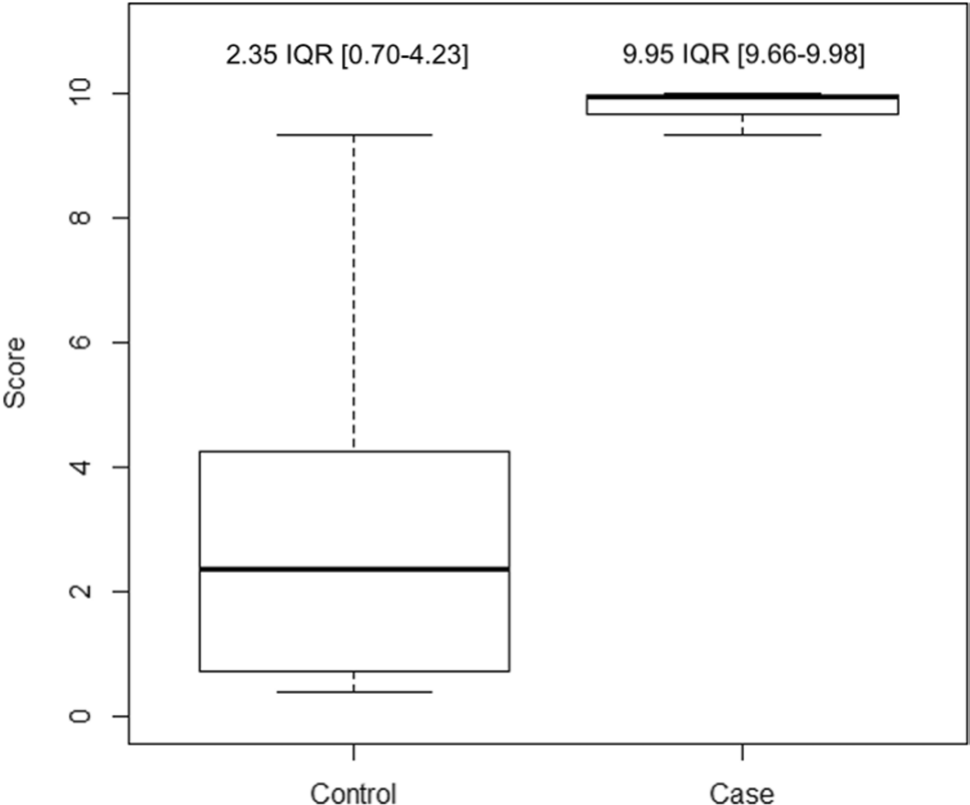
Boxplot of the score distribution among the study population according to the radioiodine refractory status

We then constructed a score to predict the probability for a patient with DTC of becoming RAIR. The score took into account all the variables of the multivariate conditional logistic regression model and ranged from 0 to 10, 10 being the highest risk of becoming RAIR. Its AUC was 0.95 (CI 0.93–0.97) (Fig. 2). The score distribution between the two groups is presented in Fig. 3. The median score was significantly higher in the RAIR-TC patients with 9.95 (IQR = 9.66–9.98) than in the radioiodine-avid group with 2.35 (IQR = 0.7–4.23) (*P* < 0.001).

The Youden’s statistic identified the optimal cut-off value for discrimination between the two groups, which was 8.9. This value was used to calculate the sensitivity, specificity, positive predictive value and negative predictive value of the scoring system, respectively, 86% (CI 80–91%), 92% (CI 90–94%), 69% (CI 62–76%), 97% (CI 95–98) (Table 4). In other words, a patient with a score higher than 8.9 had a 69 out of 100 chance of becoming RAIR, while a patient with a score lower than 8.9 had a 97 out of 100 chance of not becoming RAIR. The online calculation of our predictive score is available at https://am-my.shinyapps.io/predictive_score_of_RAIR-TC and is presented in Supplementary Material Fig. S1.

**Table 4:** Performance of the scoring system

	Value	95% CI
Sensitivity	0.86	0.80–0.91
Specificity	0.92	0.90–0.94
Positive predictive value	0.69	0.62–0.76
Negative predictive value	0.97	0.95–0.98
Area under the curve	0.95	0.93–0.97

CI, confidence interval

## Discussion

Our study included 159 patients with RAIR-TC and 759 matched-controls. The characteristics of the patients with RAIR-TC were as expected according to previous literature [7, 8], with lungs being the most frequent metastatic location followed by neck and bone metastases. Men (38%) and follicular cancers (28%) were over-represented in comparison with the whole DTC population in the literature [19]. The large number of RAIR patients included in our study allowed the identification of seven independent clinical correlated factors for the evolution towards RAIR-disease, which were: age at diagnosis ≥ 55 years; synchronous metastases at initial work-up in neck, lungs and bones; metastatic persistence or recurrence in neck and lungs.

The strength of our work relied on the original and powerful methodology. First, the nested case–control study design made it possible to control potential confusion bias as the radioiodine sensitive patients were comparable to the cases in terms of management, including the use of radioiodine, follow-up as they came from the same cohort of patients. Furthermore, matching the cases and the controls on main criteria such as patient sex, histological type and tumor size, made it possible to investigate other important potential risk factors that otherwise could have been hidden. Second, our methodology took into account the time between DTC and RAIR diagnosis in the analysis. Indeed, as RAIR-disease can occur from the beginning of patient management to 29 years later, comparing RAIR and radioiodine sensitive patients with the same duration of follow-up is an important factor in the methodology. This is the major limitation of all previously published studies on the same topic and predictive factors for becoming RAIR can, therefore, be misinterpreted. Third, our study is one of the first to have investigated the role of both synchronous and metachronous distant metastases in predicting the occurrence of RAIR status.

Furthermore, very few studies have addressed the question of risk factors for developing RAIR-disease in DTC [13–16] and the methodology as well as the choice of the control was disputable. Li et al. included 112 cases and 224 unmatched controls randomly selected among patients who had undergone total thyroidectomy and RAI therapy. The independent risk factors for developing RAIR-disease in this work were smoking, tumor histological type (FTC), ETE, number of lymph node metastases (≥ 4), lymph node metastases ratio ≥ 53% and pN stage (N1) while age and multifocality were not independent risk factors [13]. Shobab et al. studied DTC patients with DM and with a minimum follow-up of 3 years. This study included 54 RAIR-TC for 22 sex and age-matched controls and found no statistically significant differences between the RAIR and the controls in tumor size, ETE and histology [15]. Only the cumulative RAI dose and number of RAI-therapies were significantly higher in the cases. Liu et al. in a recent retrospective study focused on DTC with DM evaluated by ^18^F-FDG PET/CT. They included 223 RAIR-TC and 181 non RAIR-DTC and found that age at diagnosis ≥ 48, recurrence between the thyroidectomy and the first RAI-therapy (mean time between thyroidectomy and RAI-therapy of 9 and 26 months, respectively, for the radioiodine sensitive and RAIR groups) and ^18^FDG uptake on the metastatic site were independently associated with RAIR-DTC [16]. However, RAIR-DTC and non RAIR-DTC were not matched in this study and the median time of follow-up was short (20 months).

A meta-analysis by Luo et al. in 2020 including 13 studies concluded that ETE and high-risk histological type (including tall cell PTC, diffuse sclerosing variant of PTC, hobnail PTC, FTC and PDTC) increased the risk of developing RAIR-disease [14]. They found no difference regarding sex, age, tumor size, multifocality, or lateral lymph node metastases. However, the 13 studies included in the meta-analysis were significantly heterogeneous in terms of methodology and studied population. Some studies had a small sample size (*n* = 40 to 336) and others had a low level of evidence as they were not case–control studies but case series with unmatched controls [13, 20]. Finally, Kersting et al. focused on the subgroup of PDTC and found that tumor size > 40 mm, ETE and age > 55 were significant predictors of RAIR-disease in a total of 51 patients [21].

Based on histological and clinical variables of interest, we proposed a score for predicting the occurrence of RAIR-disease calculable after total thyroidectomy and RAI therapy with a sensitivity of 86% (CI 80–91%) and a specificity of 92% (CI 90–94%). Owing to the statistical methodology used, as cases and controls were matched on follow-up duration, the time to onset of RAIR-disease prediction was not possible.

In the literature, two prediction scores of RAIR-disease are available based on clinical and histological factors identified to be independent risk factors [13, 16]. The first one proposed by Li et al., discussed above, and including smoking, tumor histological type (FTC), ETE, number of lymph node metastases (≥ 4), lymph node metastases ratio ≥ 53%, and pN stage (N1) had an AUC of 0.876 with a sensitivity and specificity of 77.7% and 81.2% [13]. The second one proposed by Liu et al. and including age ≥ 48, recurrence between the operation and iodine-131 treatment, ^18^F-FDG uptake on the metastatic sites had an AUC of 0.898 with a sensitivity and specificity of 76% and 93% [16]. In a different way, a biological prediction model based on the preoperative thyroglobulin (pre-TG) was proposed by Cheng et al. including 90 RAIR-TC and 786 controls [22]. They found that elevated pre-TG was correlated with RAIR-disease. A cut-off value of 70.05 ng/ml was retained to distinguish between both groups with 62.2% sensitivity and 77.1% specificity. AUC was 0.76. In total, with an AUC of 0.95 and a maximal Youden index of 0.78, our model has the strength of efficiently and clearly discriminating between radioiodine sensitive and RAIR-TC patients, compared to previous scores.

Nevertheless, our work has some limitations. Matching the cases and the controls on few key criteria offered the advantages described above, but a classical cohort study design could have allowed us to adjust these factors for the whole cohort. Another limitation laid in not considering histological subtypes, especially aggressive subtypes that were not systematically available on the histology reports for our patients. At last, we could not test our score on an external validation cohort due to the rarity of the RAIR-disease. However, the results obtained by cross-validation (AUC = 0.93, maximum absolute calibration error = 0.46, Brier score = 0.16) and by bootstrap (AUC = 0.95, maximum absolute calibration error = 0.45, Brier score = 0.16) showed high internal validity of the score.

Genotyping RAIR-TC in our cohort is the subject of ongoing research. Some previous works have specifically examined the impact of mutations in the telomerase reverse transcriptase (TERT) promoter and in the BRAF oncogene, belonging to the MAPK pathway, especially the V600E mutation. While the association of BRAF and TERT mutations seems to be clearly associated with RAIR-disease [20, 23, 24], the impact of each mutation alone remains subject to debate [20, 23, 24]. TC somatic genotyping will probably help to better predict the evolution toward RAIR status.

## Conclusion

This case–control study provides the clinical characterization of a RAIR-TC population and found several independent risk factors predicting RAIR-TC, including age at diagnosis ≥ 55, vascular invasion, local and distant metastases at initial TC diagnosis and during the follow-up. A predictive score of RAIR-disease, based on clinical factors routinely available for patients treated and followed up for DTC, has been developed with high sensitivity and specificity. This available online scoring system and the identification of independent predictors of RAIR-disease may allow the optimization of long-term patient management. Early identification of high-risk patients being diagnosed with a RAIR-TC will help clinicians to rationalize RAI treatment indication or indicate redifferentiating strategy with molecular targeted therapies before RAI therapy.

### Supplementary Information

Below is the link to the electronic supplementary material.

Supplementary file 1 (DOCX 178 kb)

## Data Availability

The detailed data used to support the fndings of this study are available from the corresponding author upon request.
