# Supplementary material for: Clinico-pathological factors associated with radioiodine refractory differentiated thyroid carcinoma status
Source: J Endocrinol Invest. 2024 Apr 5;47(6):1573–81. doi: 10.1007/s40618-024-02352-z (PMC11143047; doi:10.1007/s40618-024-02352-z)
Supplement: Supplementary file 1 — (DOCX 178 kb) [file 40618_2024_2352_MOESM1_ESM.docx]

**Supplementary figure**

# Submission for journal of endocrinological investigation

# Title:

Clinico-pathological factors associated with radioiodine refractory differentiated thyroid carcinoma status.

# Authors:

Louis Schubert (MD) ^1^, Ariane Murielle Mbekwe Yepnang (PhD) ^2^, Johanna Wassermann (MD, PhD) ^3^, Yasmin Braik-Djellas (MD) ^1^, Loïc Jaffrelot (MD) ^3^, Fabiana Pani (MD, PhD) ^1^, Gabrielle Deniziaut (MD) ^4^, Charlotte Lussey-Lepoutre (MD, PhD) ^5,6^, Nathalie Chereau (MD) ^7^, Laurence Leenhardt (MD, PhD) ^1^, Marie-Odile Bernier (MD, PhD) ^2^, Camille Buffet (MD, PhD)^1,8^.

# Affiliations:

^1^ Thyroid Diseases and Endocrine Tumors Department, Sorbonne Université, Groupe de Recherche Clinique n°16, GRC Tumeurs Thyroïdiennes, AP-HP, Hôpital Pitié-Salpêtrière, 75013 Paris, France.

^2^ Laboratory of Epidemiology, Institut de Radioprotection et de Sureté Nucléaire, BP 17 92262 Fontenay aux Roses, France

^3^ Medical Oncology Department and Thyroid and Endocrine Tumors Department, Groupe de Recherche Clinique n°16, GRC Tumeurs Thyroïdiennes, AP-HP, Hôpital Pitié-Salpêtrière, 75013 Paris, France.

^4^ Pathology Department, Sorbonne Université, Groupe de Recherche Clinique n°16, GRC Tumeurs Thyroïdiennes, AP-HP, Hôpital Pitié-Salpêtrière, 75013 Paris, France.

^5^ Nuclear Medicine Department, Sorbonne Université, Groupe de Recherche Clinique n°16, GRC Tumeurs Thyroïdiennes, AP-HP, Hôpital Pitié-Salpêtrière, 75013 Paris, France.

^6^ PARCC-Inserm U970, 56 rue leblanc, 75015 Paris, France

^7^ Department of Endocrine Surgery, Sorbonne Université, Groupe de Recherche Clinique n°16, GRC Tumeurs Thyroïdiennes, AP-HP, Hôpital Pitié-Salpêtrière, 75013 Paris, France.

^8^ Laboratoire d’Imagerie Biomédicale (LIB), Sorbonne Université, CNRS UMR 7371, INSERM U1146, Paris, France

Corresponding author:

Dr Camille BUFFET

Postal address : Sorbonne Université, GRC n°16, GRC Tumeurs Thyroïdiennes
Service des Pathologies Thyroïdiennes et Tumorales Endocrines, AP-HP, Hôpital Pitié-Salpêtrière, 45-83 boulevard de l’Hôpital, 75013, Paris, France

Email address: [camille.buffet@aphp.fr](mailto:camille.buffet@aphp.fr)

Phone number: 01 84 82 77 59


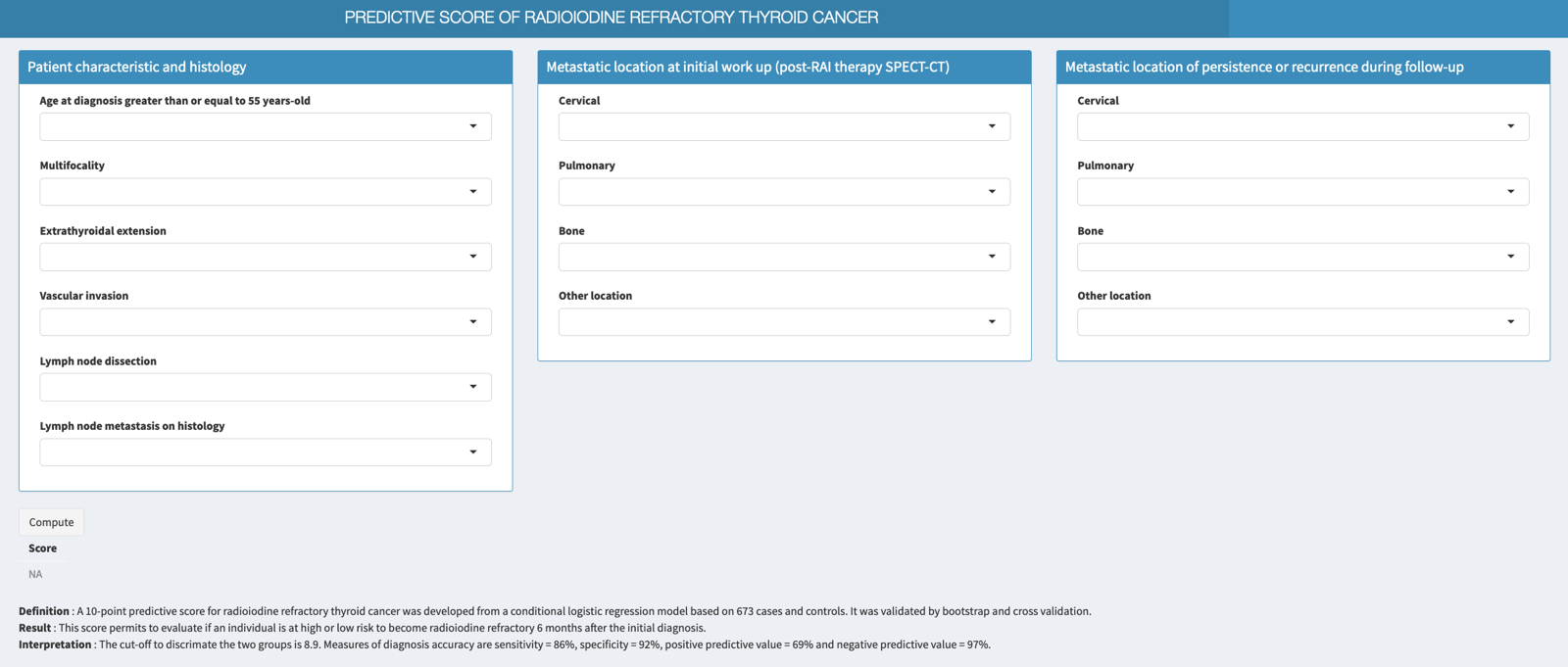


WBS, whole-body-scan.

**Fig.S1** Website for online calculation of the predictive score of developing RAIR-TC
